# Elucidating the Interaction of CF Airway Epithelial Cells and Rhinovirus: Using the Host-Pathogen Relationship to Identify Future Therapeutic Strategies

**DOI:** 10.3389/fphar.2018.01270

**Published:** 2018-11-07

**Authors:** Kak-Ming Ling, Luke W. Garratt, Timo Lassmann, Stephen M. Stick, Anthony Kicic, WAERP, AusREC, Australian Respiratory Early Surveillance Team for Cystic Fibrosis

**Affiliations:** ^1^Paediatrics, Medical School, Faculty of Healthy and Medical Science, University of Western Australia, Nedlands, WA, Australia; ^2^Telethon Kids Institute, University of Western Australia, Nedlands, WA, Australia; ^3^Department of Respiratory Medicine, Princess Margaret Hospital for Children, Perth, WA, Australia; ^4^Centre for Cell Therapy and Regenerative Medicine, School of Medicine and Pharmacology, University of Western Australia, Nedlands, WA, Australia; ^5^Occupation and Environment, School of Public Health, Curtin University, Bentley, WA, Australia; ^6^Robinson Research Institute, University of Adelaide, Adelaide, SA, Australia; ^7^Hunter Medical Research Institute: Priority Research Centre for Asthma and Respiratory Disease, New Lambton Heights, NSW, Australia; ^8^Murdoch Children's Research Institute, Melbourne, VIC, Australia; ^9^Department of Paediatrics, University of Melbourne, Melbourne, VIC, Australia

**Keywords:** cystic fibrosis, airway epithelium, rhinovirus, innate immune response, therapy, transcriptomic

## Abstract

Chronic lung disease remains the primary cause of mortality in cystic fibrosis (CF). Growing evidence suggests respiratory viral infections are often more severe in CF compared to healthy peers and contributes to pulmonary exacerbations (PEx) and deterioration of lung function. Rhinovirus is the most prevalent respiratory virus detected, particularly during exacerbations in children with CF <5 years old. However, even though rhinoviral infections are likely to be one of the factors initiating the onset of CF lung disease, there is no effective targeted treatment. A better understanding of the innate immune responses by CF airway epithelial cells, the primary site of infection for viruses, is needed to identify why viral infections are more severe in CF. The aim of this review is to present the clinical impact of virus infection in both young children and adults with CF, focusing on rhinovirus infection. Previous *in vitro* and *in vivo* investigations looking at the mechanisms behind virus infection will also be summarized. The review will finish on the potential of transcriptomics to elucidate the host-pathogen responses by CF airway cells to viral infection and identify novel therapeutic targets.

## Respiratory infections in the cystic fibrosis lung

Chronic obstructive lung disease remains the primary cause of mortality and morbidity in CF (Cutting, 2015). The defective function of the Cystic Fibrosis Transmembrane Conductance Regulator (*CFTR*) gene initiates a lifelong cycle of neutrophilic inflammation, progressive bronchiectasis, mucus obstruction and recurrent microbial infection of the CF airway. These processes typically begin in the first years of life and lead to eventual lung failure during early adulthood. The CF airway environment is vulnerable to colonization by particular bacterial and fungi species including *Haemophilus influenzae, Staphylococcus aureus, Aspergillus fumigatus*, and *Pseudomonas aeruginosa* (Gangell et al., 2011). Infection by these common pathogens typically trigger neutrophilic responses, however, these fail to eradicate the infection and lead to a sustained release of oxidants and proteases, particularly neutrophil elastase (NE) (Hartl et al., 2007; Painter et al., 2008). This neutrophil-based inflammation has been associated with the progression of structural abnormalities specifically bronchiectasis and air trapping, from as early as 3 months of age (Mott et al., 2012; Sly et al., 2013).

In addition to colonization by bacteria and fungi, the CF airway will be infected with respiratory viruses and viral infections are a major cause of PEx in the pediatric CF population (Goffard et al., 2014; Dijkema et al., 2016). The significance of viral infections in CF has been identified by the advancements in molecular diagnostic technologies to detect virus (Wat, 2015). The prevalence of respiratory viruses during CF PEx can vary from 5% up to 60% (Billard et al., 2017) and include; rhinovirus (RV), influenza A and B, respiratory syncytial virus (RSV), parainfluenza (PIV; Type 1–4), metapneumovirus, coronavirus and adenovirus (Waters and Ratjen, 2015; Flight and Jones, 2017). Earlier work suggested influenza viruses (A & B) (Pribble et al., 1990; Hiatt et al., 1999) and RSV (Abman et al., 1991; Armstrong et al., 1998) were the major cause of PEx in CF. However, studies utilizing more sensitive virological methods in the last 5 years have comprehensively established RV as the most common respiratory virus detected in CF airway (Burns et al., 2012; Wark et al., 2012; Kieninger et al., 2013; Etherington et al., 2014; Dijkema et al., 2016; Stelzer-Braid et al., 2017). Despite numerous studies into the virology of CF airways, the mechanistic link between virus infection, airway inflammation and structural lung disease remains largely unknown. Further investigation into the interaction of these disease components is warranted.

## Impact of RV infection in CF lung

A member of the *Picornaviridae* family within the Enterovirus genus, RV features a positive sense single stranded RNA genome ~7.2 kb in length. The airway epithelium is the primary site of RV infection and replication (Vareille et al., 2011). As reviewed by Palmenberg and Gern (2015), 11 viral proteins form the non-enveloped icosahedral structure. The external capsid proteins comprise of VP1, VP2, VP3, while VP4 is located between the interface of capsid protein and RNA genome. These capsid proteins feature a high degree of heterogeneity and consequently the significant antigenic diversity among RV has precluded vaccine development (Glanville and Johnston, 2015; Lewis-Rogers et al., 2017). Currently, there are more than 150 serotypes of RV, which have been classified into 3 species; RV-A, RV-B, and RV-C. Within RV-A and RV-B, strains are clustered into major and minor RV groups based upon their specificity for the intracellular adhesion molecule (ICAM-1) receptor or low-density lipoprotein receptor (LDLR), respectively (Palmenberg, 2017). Recently, the cell receptor for RV-C species has been putatively identified as cadherin related family member 3 (CDHR3), whose expression is largely confined to ciliated cells (Bochkov et al., 2015; Griggs et al., 2017; Palmenberg, 2017).

Rhinovirus infections occur all year round and children experience on average six to eight episodes per year (Worrall, 2011). Although the “common cold” is largely self-limiting, it still poses a burden on the activity and productivity of the general population (Stein, 2017). Additionally, RV infection has a more pronounced effect on vulnerable individuals such as children with CF, as summarized in Table 1. These include increased PEx (Asner et al., 2012), more severe respiratory symptoms (Burns et al., 2012; Wark et al., 2012), greater inflammation (Kieninger et al., 2013), reduced quality of life and hospitalization and prolonged antibiotic treatment (Smyth et al., 1995). Prevalence and symptoms of RV infection in patients with CF can vary between cohorts (reviewed by Billard et al., 2017), with some reporting similar rates of RV detection in both children with and without CF (de Almeida et al., 2010; Esposito et al., 2014), while others have reported significant correlations with disease progression in those with CF (Hiatt et al., 1999; van Ewijk et al., 2005). Other features such as age preference, RV serotype, viral load, impact on lung function were assessed in several studies. Susceptibility to particular RV serotype in children with CF requires further investigation due to inconsistent observations (de Almeida et al., 2010; Shah et al., 2015). RV load has been observed to be significantly higher in children with CF (>100 times) when compared to healthy controls and children with asthma (>10 times; Kieninger et al., 2013). This study also illustrated that viral load was negatively correlated to pulmonary function (Kieninger et al., 2013). Cousin et al. (2016) observed that RV-induced PEx in children with CF resulted in failure of pulmonary function recovery for up to 6 weeks. An age preference for RV-associated CF exacerbations has also been reported for young children <5 years old (Stelzer-Braid et al., 2017). However, several other studies have detected a higher frequency of RV in upper and lower airway of adults with CF via screening of sputum and throat swabs (Etherington et al., 2014; Goffard et al., 2014). Adults with CF who have viral associated PEx have been shown to have worse lung function and require more days of intravenous antibiotic treatment (Flight et al., 2014; Goffard et al., 2014). Others have also reported that adult patients who are less responsive to treatment are re-admitted for a subsequent exacerbation within a shorter time frame (Etherington et al., 2014). Finally, Flight et al. (2014) found that RV infection in adults is accompanied by an increased risk of PEx, prolonged antibiotic prescription, higher respiratory symptom scores and heightened level of C-reactive protein. As RV has a large clinical impact on those with CF, it is critical to elucidate how this virus alters host antiviral and inflammatory responses.

**Table 1:** Summary of clinical studies and outcomes related to Human Rhinovirus infection in CF.

**References**	**Cohorts**	**Sample Type**	**Virus Detection**								**Clinical Data**		
			**Positive Samples**	**Detection Method**	**RV (%)**	**Influenza (%)**	**RSV (%)**	**Parainfluenza (%)**	**Adenovirus (%)**	**Others**	**FEV1**	**Antibiotic**	**Hospitalization**
Smyth et al., 1995	CF patients (mean age 7.9 years)	Nasopharyngeal Aspirate, Serum Specimen	44/157	Virus Immunofluorescene, Culture, Serology and PCR	58	12	9	12	9	NR	Higher than other virus induced exacerbation	Prolonged treatment	No Difference
Collinson et al., 1996	CF patients (median age 7.3 years)	Nasopharyngeal Specimens	51/119	Virus Culture, PCR	41	NR	NR	NR	NR	NR	Significantly lower	More oral and intravenous antibiotic treatments for those with more infection annually	No Difference
Armstrong et al., 1998	80 infants diagnosed with CF before 12 months of age (31 infatns were hospitalized for persistent respiratory symptoms	BALs/ Nasopharyngeal Samples	14/26	Virus Immunofluorescene, Culture	14.3	14.3	43	28.5	NR	NR	NR	Not for virus infection	Higher
Hiatt et al., 1999	22 infants <2 years of age with CF (30 patient-seasons) and 27 age-matched controls (28 patient-seasons) participated	Nasopharyngeal samples	26/150	Serology, Culture Inoculation	NR	30	23	17	17	27% Picornavirus	NR	NR	RSV infection CF infants has higher rate of hospitalization
Olesen et al., 2006	75 children (media age 8)	Sputum, Laryngeal aspirations	96/606	PCR	87	3	2	6	2	NR	Significantly lower (when excluding HRV infection)	8 patients received antibiotic treatments	NR
Wat et al., 2008	71 CF patients (median age 9)	Nasal swabs and sputum samples	63/138	NASBA	15.9	15.2	2.9	10.9	NR	1% Coronavirus, 36.2% Any	NR	NR	NR
de Almeida et al., 2010	103 CF patients (median age 8.9)	Nasopharyngeal aspirates and nasal mucus specimens, sputum and oropharyngeal samples	203/408	PCR	34.1	1.2	3.7	0.6	0.2	5.9% Enterovirus, 5.6% Human Bocavirus, 4.7 Human Coronavirus, 0.7% Human metapneumovirus	NR	NR	NR
Asner et al., 2012	112 CF patients	Mid-turbinate swabs, sputum, throat swab	26/43	Immunofluroescene, multiplex PCR	23	7.6	35	15.4	11.5	34.6% Coxsackie/echovirus, 15.4% Coronavirus, 7.7% Human Metapneumovirus	No Difference	No Difference	No Difference
Stelzer-Braid et al., 2012	37 Participants (median age of 11.4) with CF	Nasal swabs and sputum samples	17/37	Multiplrc PCR	35	2.7	2.7	10.8	NR	2.7% Metapneumovirus; 46% has more than one viral or bacteria pathogen	NR	NR	NR
Kieninger et al., 2013	299 Children (median age 8.2). 195 children with CF (88 stable, 107 exacerbation), 40 children with Non CF Bronchiectasis, 29 children with Asthma and 35 Control Subjects	BALs	73/299	PCR	24.4	NR	NR	NR	NR	NR	Inversely associated with RV load	Increase use of antibiotic when increase respiratory symptoms were recorded	NR
Goffard et al., 2014	46 patients (median age of 29)	Sputum	16/64	PCR	24	3	3	3	NR	8% Coronovirus	No Difference	No Difference	NR
Esposito et al., 2014	47 CF patients with acute pulmonary exacerbation (median age of 16.7) and 31 CF patients in stable clinical condition (median age of 17.3)	Nasopharyngeal Swabs	23/78	PCR	61	17.4	4.3	NR	NR	8.6% Bocavirus, 4.3% Metapeumovirus, 4.3% Enterovirus	No Difference	NR	NR
Etherington et al., 2014	180 patients participated ion treatment with intravenous antibiotics for an acute pulmonary exacerbation. 42 patients (media age 26.5) with positive viral detection	Viral Throat Swabs	42/432	PCR	69	19	2.4	4.8	2.4	2.4% Metapneumovirus	Significantly Lower	Intravenous antibiotic for longer period	NR
Flight et al., 2014	100 adults with CF (median age of 28)	Sputum, Nose Swabs and Throat Swabs	191/626	PCR	72.5	6.1	2	2.5	4.1	13.2% Human Metapneumovirus	Lower acute fall in FEV1	Increase number of prescription	NR
Dijkema et al., 2016	20 Children with CF (0–7 years) and age matched healthy control	Nasopharyngeal Swabs	161/352 (only HRV was tested)	Nested PCR, Southern Blotting and Sequencing	45.7	NR	NR	NR	NR	NR	NR	Increase use of antibiotic prophylaxis	NR
Stelzer-Braid et al., 2017	110 children with CF	upper (nasal swab, oropharyngeal suction, and sputum) and lower (bronchoalveolar washings) respiratory tract	59/263 (<5 years old); 23/202 (older children) only HRV was tested	PCR, Nested PCR	43% (<5 years old); 12% older children	NR	NR	NR	NR	NR	NR	NR	NR

## Airway epithelium and RV infection in CF

A pseudostratified epithelium lines the surface of the lung (trachea, primary bronchi, secondary bronchi, tertiary bronchi, and bronchioles) and is composed of several cell types including ciliated cells, basal cells, secretory cells and goblet cells. These airway epithelial cells form the first point of contact with inhaled environmental insults, including respiratory viruses. To provide a physical barrier against particulates/pathogens from entering the lung tissue, numerous cell-cell connections are formed including tight junctions, adherent junctions, gap junctions, and desmosomes (Whitsett and Alenghat, 2015). To clear inhaled particles/pathogens, intraepithelial goblet cells, and submucosal glands mucous cells secret mucins. These large glycoproteins bind matter including microbes and allows effective cough clearance by the mucociliary escalator (Foster, 2015). Mucins are transported from the bronchioles to the trachea via beating cilia, expressed by airway epithelial cells of the luminal airway surface (Ma et al., 2018).

Perhaps more significantly for viral pathogens, the airway epithelium plays a crucial role in innate immunity. It has been suggested that the inflammatory responses induced by airway epithelial cells give rise to associated clinical symptoms (Jacobs et al., 2013). RVs can disrupt epithelial tight junctions including zona occludens 1 (ZO-1) protein by stimulating the production of reactive oxygen species (ROS) during viral replication (Unger et al., 2014). Work utilizing airway epithelial cells *in vitro* have shown reduced expression of other tight junction proteins, loss of epithelial integrity, disruption of extracellular matrix and subepithelial fibrosis and induction of proangiogenic molecules which enhance angiogenesis and airway remodeling (Bossios et al., 2005; Leigh et al., 2008; Bochkov et al., 2010; Tacon et al., 2010; Yeo and Jang, 2010; Looi et al., 2018).

The uptake of RV via clathrin-dependent or -independent endocytosis or through micropinocytosis occurs when RV binds to its specific receptors. Upon binding and in a low-pH environment, uncoating of RVs occurs and the virus undergoes conformational changes. The loss of the protein capsid protein VP4, and the externalization of the hydrophobic N-terminal of VP1, facilitates RVs to cross the host cell membrane (Jacobs et al., 2013; Blaas and Fuchs, 2016). Following viral uncoating and membrane rupture, RV “pathogen-associated molecular patterns” (PAMP) are recognized by the host cell via interaction with pattern recognition receptors (PRRs) including; Toll like receptors (TLRs), C-type lectin receptors (CLRs), retinoic acid-inducible gene 1 (RIG-I)-like receptors (RLRs), and nucleotide-binding oligomerization domain-like receptors (NLRs). The signaling pathways induced by TLRs and RLRs are typically host defense antiviral pathways as well as the production of antiviral substances, namely IFNs, B-defensins (Proud et al., 2004), and nitric oxide (Sanders et al., 1998). The airway epithelium also responds to RV infection by activating pro-inflammatory signaling pathways which trigger the release of chemokines and cytokines including IL-8, RANTES/CCL5, and granulocyte-macropahge colony-stimulating factor (GM-CSF), that in turn recruit neutrophils, esoinophils, natural killer cells (NK cells), and macrophages to the infected tissue. IL-6 has an important role in innate immune responses induced by RV infection and IL-6 production has been shown to be inversely correlated to cold symptoms scores and disease severity (Doyle et al., 2010). IL-15 exerts important antiviral and cytotoxic effects and is involved in the activation, differentiation, survival and recruitment of NK cells and CD8+ T cells (Jayaraman et al., 2014). IL-8 has been associated with RV infection as well as cold symptom scores (Gern et al., 2002). Furthermore, it has also been associated with neutrophilic infiltration in sputum (Gern et al., 2000). Taken together, it is evident that innate immune signaling induced by the airway epithelium is essential for effective antiviral responses.

However, in many chronic airway diseases including CF, antiviral responses are defective. Due to the pre-existing genetic defect, normal functions of the CF airway epithelium are often disrupted. As the primary site for virus entry and replication during viral infection, understanding the consequence that lack of CFTR function has on pathophysiology during virus infection is critical for effective disease management. Relevant *in vitro* experimental studies investigating RV infection in CF epithelium have been summarized in Table 2. Most studies performed to date assessed cells obtained from adult CF cohorts who had significant disease and structural lung damage. These studies report similar levels of interferon production post infection despite higher viral load being detected (Chattoraj et al., 2011; Dauletbaev et al., 2015). Studying cells from pediatric CF cohorts may generate more relevant data and potentially reveal new insights into early life RV infections that could be exploited therapeutically. Also important is the level of pro-inflammatory cytokines produced by CF epithelium following RV infection. Many studies have reported similar level of IL-8, IL-6, type I, and III IFN production, while others reported higher level of production dependent on virus strain and infectious titer (Table 2). These contradictory observations may be due in part to the age of patients involved, disease severity, RV strain, dose, and length of infection. Most studies to date including ours have focused on specific host response targets at the gene or protein level which might not reflect the global innate immune changes during RV infection. The translation of such a targeted approach would be the identification of a single molecule to address a single pathway and ultimately target one downstream effect such as the production of a single cytokine. However, knowing that the interaction of RV and the airway is multifaceted, an alternative approach that addresses this complexity is needed.

**Table 2:** Summary of *in vitro* studies of Human Rhinovirus infection in CF Airway Epithelium.

**Subject**	**Sampling Sources**	**RV Serotype**	**Inoculation Dose**	**Viral Load**	**Other Pathogens**	**Antiviral Cytokines**	**Inflammatory Cytokines**	**Apoptosis**	**References**
Adult CF (16–33 years)	BEC (ALI)	RV39	3 × 10^6^ TCID_50_	10^4^ TCID50/mL Similar to normal cells	*Pseudomonas aeruginosa* (PA)	RV: IFNβ, λ1, 2 mRNA and protein ↑ Similar to normal cells RV + PA: IFNβ, λ1, 2 mRNA and protein ↓ Only in CF cells	RV: IL8 mRNA ↑ RV + PA: IL-8 mRNA ↑↑ Similar to normal cells	Not measured	Chattoraj et al., 2011
Adult CF (19–41 years)	BEC from explant lung	RV16	MOI 0.1	>10^4^ copy number Higher in CF	*Pseudomonas aeruginosa* (PA)	RV: IFNβ mRNA↑ Similar to normal cells; OAS1 mRNA↑ Similar to normal cells	RV: IL8 mRNA ↑ Similar to normal cells	Not measured	Dauletbaev et al., 2015
Young children with CF (1–7 years)	AEC	RV1b	MOI 3, 25	~1500 copy/ng RNA	No	Not measured	IL-6 and IL-8 protein ↑ in CF cells only for infection with RV1b of MOI 25 at 48 hours	Reduced compared to normal cells	Sutanto et al., 2011
Children and adults with CF (4.5–48.9 years)	NEC, BEC and cell lines	RV16, RV1b	MOI 2	Not measured	No	Not measured	IL-6, IL-8, IP-10, MCP-1, RANTES↑ Similar to normal cells	Similar apoptosis, ↑necrosis compared to normal cells	Kieninger et al., 2013
Children with CF (3–11 years)	BEC	RV16, RV1b	MOI 4	Not measured	No	RV16: ↓ IFNs (IFN-λ1, IFN-λ2/3 and IFN-β), PRRs (RIG-1 and MDA-5) and ISGs (PKR, OAS1, viperin and MxA). RV1b: ↑ PRRs (TLR3 and RIG-1) and IFN pathway (IFN-λ1 and IFN-λ2/3 compared to normal cells	↑ CXCL8/IL-8, IL-6 and CXCL10/IP-10	Not measured	Schögler et al., 2014

*AEC, Airway Epithelial Cells; BEC, Bronchial Epithelial Cells; NEC, Nasal Epithelial Cells*.

## Past and current therapies

To date, there have been no studies performed that have focused on potential treatments for RV infection in CF individuals. As RV continues to be the most prevalently detected virus in the all individuals including CF airway, additional evidence is needed to specify its connection with the existing factors such as lack of CFTR and airway inflammation through molecular intermediates and cellular signaling pathways. Common anti-inflammatories including oral corticosteroids and high-dose ibuprofen are unsuitable for treatment in infants and preschool children due to their long-term side effects (Lai et al., 2000; Fennell et al., 2007). Azithromycin may have some interesting antiviral properties, specifically in reducing RV replication via amplification of the IFN pathway-mediated antiviral responses (Schögler et al., 2014). Nevertheless, clinical studies are necessary to elucidate the clinical impact of azithromycin against RV-induced PEx in patients with CF. Vaccination is an important part of CF clinical care, however vaccine development for RV has been rather challenging due to the wide range of antigenic diversity of more than 150 serotypes of RV across three different strains. Technical difficulties in producing sufficient amounts of antigen against multiple RV serotypes using animal models (as reviewed in Del Vecchio et al., 2015) also remains a challenge and as result, the development of a long-lasting RV vaccine has not been successful. This has been further compounded by the lack of a suitable model (other than human) that is fully permissive to RV infection as well as insufficient clinical data to identify and prioritize dominant RV serotypes. In addition, no suitable animal models for CF exhibit complete spectrum of CF phenotype besides CF pig and ferret which are strictly limited for research use (as reviewed in Rosen et al., 2018).

Since vaccination is unavailable, other approaches have been explored in healthy and disease cohorts other than CF. An early approach by Turner et al. (1999) aimed to prevent HRV binding to its receptors via administration of inhaled recombinant soluble ICAM-1 (Tremacamra). Although the reduction in symptom severity and viral shedding were promising, the high costs and dosing regimen recommended (6 times daily) made translation of this therapy into the clinical setting prohibitive (Turner et al., 1999). Targeting viral replication has perhaps been the highest priority in past therapeutic development, where capsid-binding drugs bind to the hydrophobic “pocket” of the viral capsid (reviewed in McKinlay et al., 1992). Pleconaril and Pirodavir were discontinued due to unforeseen side effects and drug efficacy. Reformulated Pleconaril and Vapendavir have completed clinical trials although results have yet to be published [ClinicalTrials.gov (NCT00394914 & NCT01175226)]. A recently discovered compound IMP-1088 offers more promise (Mousnier et al., 2018). This molecule was shown to inhibit human N-myristoyltransferases NMT1 and NMT2, prevent virus assembly and suppress RV replication and infection across various RV strains without inducing cytotoxicity. However, most of this work was demonstrated using cell lines or adult primary cells and further assessment of IMP-1088 on primary cells from young children with CF is necessary. The outcome of such studies would be informative as to whether this compound exerts similar efficacy across all cohorts, as more than 30 polymorphic DNA loci associated with host variation in gene expression called responsereQTLs to rhinovirus infection has been previously reported (Çalişkan et al., 2015).

Alternatively, the roles of type I IFN administration in enhancing the primary antiviral signaling pathway of innate immunity have also been assessed. Early studies involving the prophylactic administration of IFN-α2 or IFN-β were found to demonstrate a reduction in number of RV-induced episodes but no difference in symptom severity or duration (Farr et al., 1984; Hayden et al., 1986; Monto et al., 1986; Sperber et al., 1988). Multiple side effects from high dose administration of IFNs, including nasal bleeding, transient leukopenia and sore throat have also been reported (Sperber et al., 1988). A more recent study assessed low dose IFN-β administration and although antiviral activity was enhanced, it did not aid in reducing cold symptoms of viral induced exacerbations asthma cohorts (Djukanović et al., 2014). Ruuskanen et al. (2014) also suggested that short-term subcutaneous pegylated IFN-α in combination with oral ribavirin treatment rapidly decreased RV RNA in recurrent or chronic rhinovirus infection in immunocompromised patients.

Alternative therapies have not been thoroughly assessed in CF cohorts. While ongoing clinical trials are comprehensive in evaluating the efficacy of CFTR potentiators and correctors for application on mutation specific patients, improvements in infection and inflammation therapies would be highly desirable for all individuals with CF. Indeed, Ivacaftor has been found to reduce sputum *P. aeruginosa* density (>60-fold) and airway inflammation significantly (Hisert et al., 2017). Whether improvements in CFTR folding or function will enhance antiviral responses in children with CF warrant further investigation. Multi-target drug design also holds potential and could be employed to exert both antiviral and anti-inflammation effects. Understanding how host anti-viral and inflammatory responses differ in CF airways, particularly young children, is critical in facilitating the development of new therapeutic treatments that can limit CF disease progression.

## New therapeutic for RV infections

Current therapies directed at RV are mainly focusing on specific viral proteins or inhibition of viral cycle. However, some of these drugs are not effective on drug-resistant viral strains. The current review proposes an alternative approach that focuses on host cellular pathways and factors. To expedite novel therapeutic strategies, investigation on how cellular signaling pathways can be altered by RV infection and how these alterations can be manipulated by new compounds or drugs are crucial for new therapeutic development. The current field of system biology and adoption of high-throughput technologies through transcriptomics not only facilitates characterization of the host-pathogen interaction in a more comprehensive manner, but also aids in understanding how developed and repurposed compounds exert their antiviral properties on RV infection in CF patients.

Knowing viruses can manipulate the host signaling processes and thus altering the host-pathogens interactions (Christiaansen et al., 2015), evaluating the global changes in gene expression during infection via employing gene/transcriptomics could elucidate crucial messages for therapeutic target identification. Transcriptomics is used to study the total RNA output of a cell. Early transcriptomic analyses were performed using microarrays which have customized probes, while current transcriptomic analyses rely on high-throughput RNA sequencing which capture global transcriptome (Mortazavi et al., 2008). These techniques allow analysis for “all molecules” regulated at the gene level. By illustrating their interaction within the cells and the complexities of host-pathogen interactions, enhancing or diminishing specific molecules as well as precise characterization of specific targets can be a more promising therapeutic approach (as reviewed in Cesur and Durmuş, 2018). Generally, computational approaches are used to organize or manage these data sets and interpret the biological inference, including network analysis (such as InnateDB, NetworkAnlayst, and Cytoscape) and pathway analysis (such as Reactome, Kegg Pathway, and Pantherdb).

In the context of host-viral interaction, transcriptomic analysis has been successfully applied to identify the uncharacterized isoforms from wild-type dengue infected host RNA from human hepatoma cells. The authors demonstrated that infection with wild-type dengue virus elicited a different host response compared to infection with a vaccine sensitive strain, highlighting the potential of strain-specific responses (Sessions et al., 2013). Transcriptional profiling of blood specimens from symptomatic and asymptomatic patients with RV infection have revealed that individuals with active infection demonstrate a robust transcriptional signature of immune-related genes (Heinonen et al., 2016). In other disease settings including asthma and allergic rhinitis, transcriptomic responses of human respiratory cells to surrogate RV infection [Poly(I:C) stimulation] have potentially identified disease-specific signatures (Wagener et al., 2014). Therefore, it is imperative to assess the global transcript expression and investigate the host-viral interaction, given that CF is a defined genetic disease condition and live RV infection can truly represent an active infection which might involve modification of the host response. This approach is not only applied to protein-coding RNA but also provide insights to critical non-coding RNA such as short non-coding RNAs (miRNAs) and long non-coding RNAs (lncRNAs) which are key regulators for modulation of gene expression (Delpu et al., 2016).

Mapping genes on a complete network allows identification of key hub genes and central genes with high connectivity which exert large effects on signal transduction. Molecular network analysis also allows enrichment of functional modules to identify which area group of genes are cooperatively working together to perform specific biological function and could be associated with disease setting (reviewed by Csermely et al., 2013). Some examples include, the identification of 16 strongly connected hub genes as potential antifungal drug targets against *Candida albicans* (Altwasser et al., 2012), the identification of new key genes for type 1 diabetes (Safari-Alighiarloo et al., 2016) as well as certain cancers (Zaman et al., 2013; Jin et al., 2015). Moreover, omics data has discovered disease modules and revealed substantial inter-patient heterogeneity, highlighting the potential importance of customize treatments to conditions. Numerous algorithms have been introduced to identify disease modules, including ModuleDiscoverer that identified a rodent model of non-alcoholic steatohepatitis (NASH), as well as a severe form of non-alcoholic fatty liver disease (NAFLD) (Vlaic et al., 2018). To maximize the efficacy and treatment outcome, patient individual characteristics, including their genetic profile needs to be considered. Although using biological network analysis can expedite the drug discovery process, the timeline from target identification to clinic application can still be lengthy.

An alternative strategy is to explore drug repurposing. Integrated analysis of disease-gene profiles, pathway analysis, and mining of FDA approved drug databases can be carried out to identify correlations of common pathways with certain compounds or molecules at the network level. Successful examples of drug repurposing based on transcriptomic analyses include the identification of topiramate for the treatment of inflammatory bowel disease (IBD) and cimetidine for the treatment of lung adenocarcinoma (Dudley et al., 2011; Sirota et al., 2011). Using a large-scale expression signature, Lee et al. (2016) have also identified that ivermectin, trifluridine, astemizole, amlodipine, maprotiline, apomorphine, mometasone, and nortriptyline show significant anti-proliferative activity against glioblastoma. With the recent establishment of ImmPort, a data repository that promotes research dataset repurposing (Bhattacharya et al., 2018), the identification of novel targets and repurposed drugs that target these has been accelerated further. Currently, there is paucity of data in CF-related RV-therapy given its impact on CF lung disease and thus new interventions are urgently required. The strategy to repurpose already approved drugs could advance antiviral therapies by reducing cost and improving and quality of life for affected individuals.

## Conclusion

**Figure 1:**
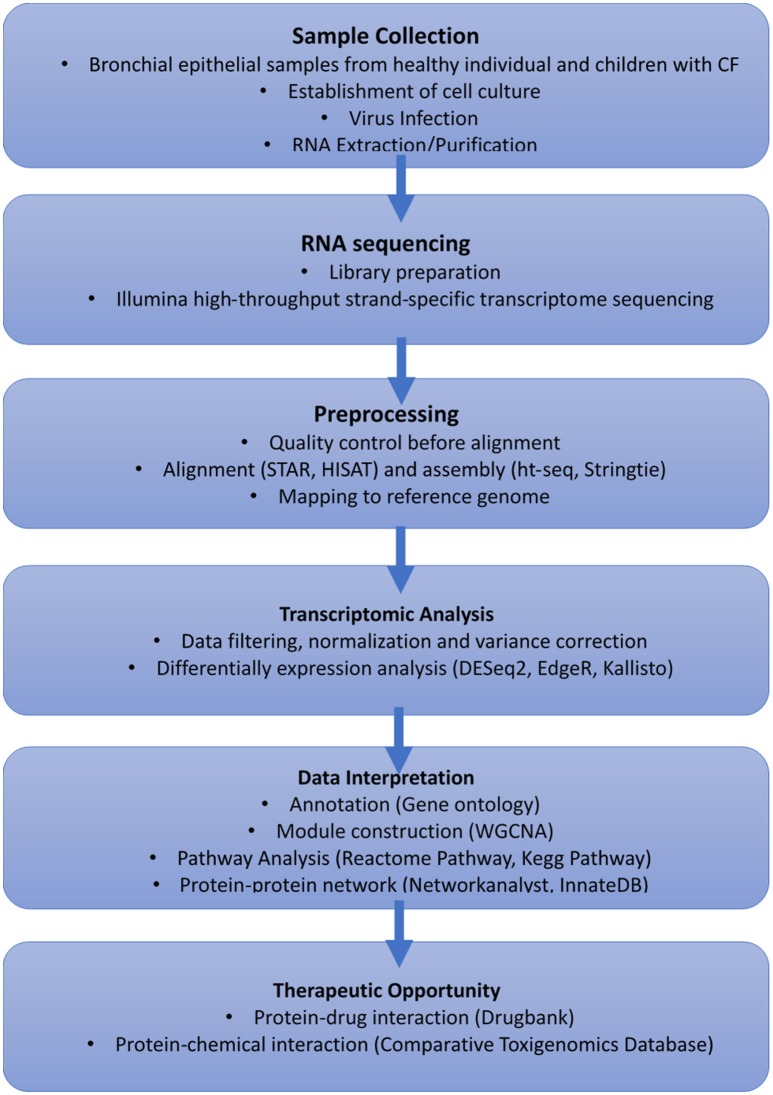
Proposed workflow using transcriptomics to elucidate future treatment for cold virus infection in CF.

RV infection remains a significant cause of pulmonary exacerbation in CF. There has been little investigation into antiviral therapies in CF especially in young children who are more susceptible to these types of infection. However, modern virological procedures and omic technologies now facilitate more in-depth studies of the genes and molecular pathways involved in aberrant CF antiviral responses to RV. We propose transcriptomics could be leveraged to elucidate future therapeutic intervention for treatment of rhinovirus infection in CF. For example, a global gene expression profile of bronchial epithelial cells from patients with CF, under baseline conditions and after RV infection will be profiled following next-generation RNA sequencing (Figure 1). Sequences can be aligned and mapped to already available reference genomes to identify differentially expressed genes pre- and post-infection. The identified genes could then be annotated using online repositories or libraries to investigate their enriched functional biological pathways. Moreover, networks or subnetworks can then be constructed by mapping identified genes to explore their relationship using curated protein-protein interaction databases. Therapeutic opportunities can also identify by exploring protein-protein interaction and protein-transcription factor, protein-drug interaction as well as chemical interaction databases. Finally, monolayer cell cultures which have previously been found to be more susceptible for RV infection (Bochkov et al., 2010) represent an oversimplified model for the multicellular interactions of epithelial (ciliated cells, goblet cells) and immune cells (dendritic cells, neutrophils). Indeed, functional validation utilizing human *in vitro* 3D airway models (Boda et al., 2018) will be needed to further elucidate to host-pathogen interactions. The emergence of single cell transcriptomics could be used to compliment 3D airway models and accelerate progress in this new era of scientific research. Overall, the advancement of these promising tools should aid in expediting new therapeutic intervention in this sphere.

## Author contributions

K-ML, LG, and AK conceptualized the contents of the manuscript. K-ML wrote the first draft of the manuscript. K-ML, LG, TL, SS, and AK contributed to the drafting and editing of the manuscript. WAERP, AusREC, and AREST CF approved the final submission of the manuscript.

### Conflict of interest statement

The authors declare that the research was conducted in the absence of any commercial or financial relationships that could be construed as a potential conflict of interest.

## References

[B1] Abman S. H. Ogle J. W. Harbeck R. J. Butler-Simon N. Hammond K. B. Accurso F. J. (1991). Early bacteriologic, immunologic, and clinical courses of young infants with cystic fibrosis identified by neonatal screening. J. Pediatr. 119, 211–217. 10.1016/S0022-3476(05)80729-2 1907318

[B2] Altwasser R. Linde J. Buyko E. Hahn U. Guthke R. (2012). Genome-wide scale-free network inference for *Candida albicans*. Front. Microbiol. 3:51. 10.3389/fmicb.2012.00051 22355294 PMC3280432

[B3] Armstrong D. Grimwood K. Carlin J. B. Carzino R. Hull J. Olinsky A.. (1998). Severe viral respiratory infections in infants with cystic fibrosis. Pediatr. Pulmonol. 26, 371–379. 10.1002/(SICI)1099-0496(199812)26:6<371::AID-PPUL1>3.0.CO;2-N 9888211

[B4] Asner S. Waters V. Solomon M. Yau Y. Richardson S. E. Grasemann H.. (2012). Role of respiratory viruses in pulmonary exacerbations in children with cystic fibrosis. J. Cyst. Fibros. 11, 433–439. 10.1016/j.jcf.2012.04.006 22579414 PMC7105203

[B5] Bhattacharya S. Dunn P. Thomas C. G. Smith B. Schaefer H. Chen J.. (2018). ImmPort, toward repurposing of open access immunological assay data for translational and clinical research. Sci. Data 5:180015. 10.1038/sdata.2018.15 29485622 PMC5827693

[B6] Billard L. Le Berre R. Pilorgé L. Payan C. Héry-Arnaud G. Vallet S. (2017). Viruses in cystic fibrosis patients' airways. Crit. Rev. Microbiol. 43, 690–708. 10.1080/1040841X.2017.1297763 28340310

[B7] Blaas D. Fuchs R. (2016). Mechanism of human rhinovirus infections. Mol. Cell. Pediatr. 3:21. 10.1186/s40348-016-0049-3 27251607 PMC4889530

[B8] Bochkov Y. A. Hanson K. M. Keles S. Brockman-Schneider R. A. Jarjour N. N. Gern J. E. (2010). Rhinovirus-induced modulation of gene expression in bronchial epithelial cells from subjects with asthma. Mucosal Immunol. 3, 69–80. 10.1038/mi.2009.109 19710636 PMC2884103

[B9] Bochkov Y. A. Watters K. Ashraf S. Griggs T. F. Devries M. K. Jackson D. J.. (2015). Cadherin-related family member 3, a childhood asthma susceptibility gene product, mediates rhinovirus C binding and replication. Proc. Natl. Acad. Sci. U.S.A. 112, 5485–5490. 10.1073/pnas.1421178112 25848009 PMC4418890

[B10] Boda B. Benaoudia S. Huang S. Bonfante R. Wiszniewski L. Tseligka E. D.. (2018). Antiviral drug screening by assessing epithelial functions and innate immune responses in human 3D airway epithelium model. Antiviral Res. 156, 72–79. 10.1016/J.ANTIVIRAL.2018.06.007 29890184 PMC7113743

[B11] Bossios A. Psarras S. Gourgiotis D. Skevaki C. L. Constantopoulos A. G. Saxoni-Papageorgiou P.. (2005). Rhinovirus infection induces cytotoxicity and delays wound healing in bronchial epithelial cells. Respir. Res. 6:114. 10.1186/1465-9921-6-114 16216126 PMC1283981

[B12] Burns J. L. Emerson J. Kuypers J. Campbell A. P. Gibson R. L. McNamara S.. (2012). Respiratory viruses in children with cystic fibrosis: viral detection and clinical findings. Influenza Other Respir. Viruses 6, 218–223. 10.1111/j.1750-2659.2011.00292.x 21955319 PMC4941093

[B13] Çalişkan M. Baker S. W. Gilad Y. Ober C. (2015). Host genetic variation influences gene expression response to rhinovirus infection. PLoS Genet. 11:e1005111. 10.1371/journal.pgen.1005111 25874939 PMC4395341

[B14] Cesur M. F. Durmuş S. (2018). Systems biology modeling to study pathogen-host interactions, in Host-Pathogen Interactions. Methods in Molecular Biology, eds Medina C. López-Baena F. (New York, NY: Human Press), 97–112. 10.1007/978-1-4939-7604-1_10 29288450

[B15] Chattoraj S. S. Ganesan S. Jones A. M. Helm J. M. Comstock A. T. Bright-Thomas R.. (2011). Rhinovirus infection liberates planktonic bacteria from biofilm and increases chemokine responses in cystic fibrosis airway epithelial cells. Thorax 66, 333–339. 10.1136/thx.2010.151431 21289024 PMC3208236

[B16] Christiaansen A. Varga S. M. Spencer J. V. (2015). Viral manipulation of the host immune response. Curr. Opin. Immunol. 36, 54–60. 10.1016/j.coi.2015.06.012 26177523 PMC4593765

[B17] Collinson J. Nicholson K. G. Cancio E. Ashman J. Ireland D. C. Hammersley V.. (1996). Effects of upper respiratory tract infections in patients with cystic fibrosis. Thorax 51, 1115–1122. 10.1136/thx.51.11.1115 8958895 PMC1090523

[B18] Cousin M. Molinari N. Foulongne V. Caimmi D. Vachier I. Abely M. (2016). Rhinovirus-associated pulmonary exacerbations show a lack of FEV 1 improvement in children with cystic fibrosis. Influenza Other Respir. Viruses 10, 109–112. 10.1111/irv.12353 26493783 PMC4746558

[B19] Csermely P. Korcsmáros T. Kiss H. J. M. London G. Nussinov R. (2013). Structure and dynamics of molecular networks: a novel paradigm of drug discovery: a comprehensive review. Pharmacol. Ther. 138, 333–408. 10.1016/j.pharmthera.2013.01.016 23384594 PMC3647006

[B20] Cutting G. R. (2015). Cystic fibrosis genetics: from molecular understanding to clinical application. Nat. Rev. Genet. 16, 45–56. 10.1038/nrg3849 25404111 PMC4364438

[B21] Dauletbaev N. Das M. Cammisano M. Chen H. Singh S. Kooi C.. (2015). Rhinovirus load is high despite preserved interferon-β response in cystic fibrosis bronchial epithelial cells. PLoS ONE 10:e0143129. 10.1371/journal.pone.0143129 26599098 PMC4658124

[B22] de Almeida M. B. Zerbinati R. M. Tateno A. F. Oliveira C. M. Romão R. M. Rodrigues J. C.. (2010). Rhinovirus C and respiratory exacerbations in children with cystic fibrosis. Emerg. Infect. Dis. 16, 996–999. 10.3201/eid1606.100063 20507756 PMC3086221

[B23] Del Vecchio A. M. Branigan P. J. Barnathan E. S. Flavin S. K. Silkoff P. E. Turner R. B. (2015). Utility of animal and *in vivo* experimental infection of humans with rhinoviruses in the development of therapeutic agents for viral exacerbations of asthma and chronic obstructive pulmonary disease. Pulm. Pharmacol. Ther. 30, 32–43. 10.1016/J.PUPT.2014.10.005 25445932 PMC7110859

[B24] Delpu Y. Larrieu D. Gayral M. Arvanitis D. Dufresne M. Cordelier P. (2016). Noncoding RNAs: clinical and therapeutic applications, in Drug Discovery in Cancer Epigenetics, eds Egger G. Arimondo P. (Academic Press), 305–326. 10.1016/B978-0-12-802208-5.00012-6

[B25] Dijkema J. S. Ewijk B. E. van Wilbrink B. Wolfs T. F. W. Kimpen J. L. L. Ent C. K. van der (2016). Frequency and duration of rhinovirus infections in children with cystic fibrosis and healthy controls: a longitudinal cohort study. Pediatr. Infect. Dis. J. 35, 379–383. 10.1097/INF.0000000000001014 26658528

[B26] Djukanović R. Harrison T. Johnston S. L. Gabbay F. Wark P. Thomson N. C.. (2014). The effect of inhaled IFN-β on worsening of asthma symptoms caused by viral infections. A randomized trial. Am. J. Respir. Crit. Care Med. 190, 145–154. 10.1164/rccm.201312-2235OC 24937476 PMC4226052

[B27] Doyle W. J. Casselbrant M. L. Li-Korotky H.-S. Doyle A. P. C. Lo C.-Y. Turner R.. (2010). The interleukin 6–174 C/C genotype predicts greater rhinovirus illness. J. Infect. Dis. 201, 199–206. 10.1086/649559 20001857 PMC2943745

[B28] Dudley J. T. Sirota M. Shenoy M. Pai R. K. Roedder S. Chiang A. P.. (2011). Computational repositioning of the anticonvulsant topiramate for inflammatory bowel disease. Sci. Transl. Med. 3:96ra76. 10.1126/scitranslmed.3002648 21849664 PMC3479650

[B29] Esposito S. Daleno C. Scala A. Castellazzi L. Terranova L. Sferrazza Papa S.. (2014). Impact of rhinovirus nasopharyngeal viral load and viremia on severity of respiratory infections in children. Eur. J. Clin. Microbiol. Infect. Dis. 33, 41–48. 10.1007/s10096-013-1926-5 23893065 PMC7088146

[B30] Etherington C. Naseer R. Conway S. P. Whitaker P. Denton M. Peckham D. G. (2014). The role of respiratory viruses in adult patients with cystic fibrosis receiving intravenous antibiotics for a pulmonary exacerbation. J. Cyst. Fibros. 13, 49–55. 10.1016/j.jcf.2013.06.004 23891398

[B31] Farr B. M. Gwaltney J. M. Adams K. F. Hayden F. G. (1984). Intranasal interferon-α2 for prevention of natural rhinovirus colds. Antimicrob. Agents Chemother. 26, 31–34. 10.1128/AAC.26.1.31 6089652 PMC179911

[B32] Fennell P. B. Quante J. Wilson K. Boyle M. Strunk R. Ferkol T. (2007). Use of high-dose ibuprofen in a pediatric cystic fibrosis center. J. Cyst. Fibros. 6, 153–158. 10.1016/j.jcf.2006.06.003 16844429

[B33] Flight W. Jones A. (2017). The diagnosis and management of respiratory viral infections in cystic fibrosis. Expert Rev. Respir. Med. 11, 221–227. 10.1080/17476348.2017.1288102 28132571

[B34] Flight W. G. Bright-Thomas R. J. Tilston P. Mutton K. J. Guiver M. Morris J.. (2014). Incidence and clinical impact of respiratory viruses in adults with cystic fibrosis. Thorax 69, 247–253. 10.1136/thoraxjnl-2013-204000 24127019

[B35] Foster W. M. (2015). Mucociliary function, in Comparative Biology of the Normal Lung, 2nd Edn., ed R. A. Parent (Academic Press), 561–579. 10.1016/B978-0-12-404577-4.00029-1

[B36] Gangell C. L. Gard S. E. Douglas T. A. Park J. de Klerk N. H. Keil T.. (2011). Inflammatory responses to individual microorganisms in the lungs of children with cystic fibrosis. Clin. Infect. Dis. 53, 425–432. 10.1093/cid/cir399 21844026

[B37] Gern J. E. Martin M. S. Anklam K. A. Shen K. Roberg K. A. Carlson-Dakes K. T.. (2002). Relationships among specific viral pathogens, virus-induced interleukin-8, and respiratory symptoms in infancy. Pediatr. Allergy Immunol. 13, 386–393. 10.1034/j.1399-3038.2002.01093.x 12485313

[B38] Gern J. E. Vrtis R. Grindle K. A. Swenson C. Busse W. W. (2000). Relationship of upper and lower airway cytokines to outcome experimental rhinovirus infection. Am. J. Respir. Crit. Care Med. 162, 2226–2231. 10.1164/ajrccm.162.6.2003019 11112143

[B39] Glanville N. Johnston S. L. (2015). Challenges in developing a cross-serotype rhinovirus vaccine. Curr. Opin. Virol. 11, 83–88. 10.1016/j.coviro.2015.03.004 25829255

[B40] Goffard A. Lambert V. Salleron J. Herwegh S. Engelmann I. Pinel C.. (2014). Virus and cystic fibrosis: rhinoviruses are associated with exacerbations in adult patients. J. Clin. Virol. 60, 147–153. 10.1016/j.jcv.2014.02.005 24637203 PMC7108260

[B41] Griggs T. F. Bochkov Y. A. Basnet S. Pasic T. R. Brockman-Schneider R. A. Palmenberg A. C.. (2017). Rhinovirus C targets ciliated airway epithelial cells. Respir. Res. 18:84. 10.1186/s12931-017-0567-0 28472984 PMC5418766

[B42] Hartl D. Latzin P. Hordijk P. Marcos V. Rudolph C. Woischnik M.. (2007). Cleavage of CXCR1 on neutrophils disables bacterial killing in cystic fibrosis lung disease. Nat. Med. 13, 1423–1430. 10.1038/nm1690 18059279

[B43] Hayden F. G. Albrecht J. K. Kaiser D. L. Gwaltney J. M. (1986). Prevention of natural colds by contact prophylaxis with intranasal alpha_2_-interferon. N. Engl. J. Med. 314, 71–75. 10.1056/NEJM198601093140202 3001519

[B44] Heinonen S. Jartti T. Garcia C. Oliva S. Smitherman C. Anguiano E. (2016). Value of host transcriptome analysis. Rhinovirus detect. Symptomatic asymptomatic child. Am. J. Respir. Crit. Care Med. 193, 772–782. 10.1164/rccm.201504-0749OC 26571305 PMC4824929

[B45] Hiatt P. W. Grace S. C. Kozinetz C. A. Raboudi S. H. Treece D. G. Taber L. H.. (1999). Effects of viral lower respiratory tract infection on lung function in infants with cystic fibrosis. Pediatrics 103, 619–626. 10.1542/peds.103.3.619 10049966

[B46] Hisert K. B. Heltshe S. L. Pope C. Jorth P. Wu X. Edwards R. M.. (2017). Restoring cystic fibrosis transmembrane conductance regulator function reduces airway bacteria and inflammation in people with cystic fibrosis and chronic lung infections. Am. J. Respir. Crit. Care Med. 195, 1617–1628. 10.1164/rccm.201609-1954OC 28222269 PMC5476912

[B47] Jacobs S. E. Lamson D. M. St George K. Walsh T. J. (2013). Human rhinoviruses. Clin. Microbiol. Rev. 26, 135–162. 10.1128/CMR.00077-12 23297263 PMC3553670

[B48] Jayaraman A. Jackson D. J. Message S. D. Pearson R. M. Aniscenko J. Caramori G.. (2014). IL-15 complexes induce NK- and T-cell responses independent of type i IFN signaling during rhinovirus infection. Mucosal Immunol. 7, 1151–1164. 10.1038/mi.2014.2 24472849 PMC4284198

[B49] Jin B. Wang W. Du G. Huang G. Z. Han L. T. Tang Z. Y.. (2015). Identifying hub genes and dysregulated pathways in hepatocellular carcinoma. Eur. Rev. Med. Pharmacol. Sci. 19, 592–601. 25753876

[B50] Kieninger E. Singer F. Tapparel C. Alves M. P. Latzin P. Tan H. L.. (2013). High rhinovirus burden in lower airways of children with cystic fibrosis. Chest 143, 782–790. 10.1378/chest.12-0954 23188200

[B51] Lai H.-C. FitzSimmons S. C. Allen D. B. Kosorok M. R. Rosenstein B. J. Campbell P. W.. (2000). Risk of persistent growth impairment after alternate-day prednisone treatment in children with cystic fibrosis. N. Engl. J. Med. 342, 851–859. 10.1056/NEJM200003233421204 10727589

[B52] Lee H. Kang S. Kim W. (2016). Drug repositioning for cancer therapy based on large-scale drug-induced transcriptional signatures. PLoS ONE 11:e0150460. 10.1371/journal.pone.0150460 26954019 PMC4783079

[B53] Leigh R. Oyelusi W. Wiehler S. Koetzler R. Zaheer R. S. Newton R.. (2008). Human rhinovirus infection enhances airway epithelial cell production of growth factors involved in airway remodeling. J. Allergy Clin. Immunol. 121, 1238.e4–1245.e4. 10.1016/j.jaci.2008.01.067 18355907

[B54] Lewis-Rogers N. Seger J. Adler F. R. (2017). Human rhinovirus diversity and evolution: how strange the change from major to minor. J. Virol. 91, e01659–e01616. 10.1128/JVI.01659-16 28100614 PMC5355621

[B55] Looi K. Buckley A. G. Rigby P. J. Garratt L. W. Iosifidis T. Zosky G. R.. (2018). Effects of human rhinovirus on epithelial barrier integrity and function in children with asthma. Clin. Exp. Allergy 48, 513–524. 10.1111/cea.13097 29350877

[B56] Ma J. Rubin B. K. Voynow J. A. (2018). Mucins, mucus, and goblet cells. Chest 154, 169–176. 10.1016/j.chest.2017.11.008 29170036

[B57] McKinlay M. A. Pevear D. C. York N. Rossmann M. G. (1992). Treatment of the picornavirus common cold by inhibitors or viral uncoating and attachment. Annu. Rev. Microbiol. 46, 635–654. 10.1146/annurev.mi.46.100192.003223 1332585

[B58] Monto A. S. Shope T. C. Schwartz S. A. Albrecht J. K. (1986). Intranasal interferon-alpha 2b for seasonal prophylaxis of respiratory infection. J. Infect. Dis. 154, 128–133. 10.1093/infdis/154.1.128 3011917 PMC7109820

[B59] Mortazavi A. Williams B. A. McCue K. Schaeffer L. Wold B. (2008). Mapping and quantifying mammalian transcriptomes by RNA-Seq. Nat. Methods 5, 621–628. 10.1038/nmeth.1226 18516045 PMC13303166

[B60] Mott L. S. Park J. Murray C. P. Gangell C. L. De Klerk N. H. Robinson P. J.. (2012). Progression of early structural lung disease in young children with cystic fibrosis assessed using CT. Thorax 67, 509–516. 10.1136/thoraxjnl-2011-200912 22201161

[B61] Mousnier A. Bell A. S. Swieboda D. P. Morales-Sanfrutos J. Pérez-Dorado I. Brannigan J. A.. (2018). Fragment-derived inhibitors of human N-myristoyltransferase block capsid assembly and replication of the common cold virus. Nat. Chem. 10, 599–606. 10.1038/s41557-018-0039-2 29760414 PMC6015761

[B62] Olesen H. V. Nielsen L. P. Schiotz P. O. (2006). Viral and atypical bacterial infections in the outpatient pediatric cystic fibrosis clinic. Pediatr. Pulmonol. 41, 1197–1204. 10.1002/ppul.20517 17058280

[B63] Painter R. G. Bonvillain R. W. Valentine V. G. Lombard G. A. LaPlace S. G. Nauseef W. M.. (2008). The role of chloride anion and CFTR in killing of *Pseudomonas aeruginosa* by normal and CF neutrophils. J. Leukoc. Biol. 83, 1345–1353. 10.1189/jlb.0907658 18353929 PMC2901559

[B64] Palmenberg A. C. (2017). Rhinovirus C, asthma, and cell surface expression of virus receptor CDHR3. J. Virol. 91:e00072–17. 10.1128/JVI.00072-17 28100615 PMC5355607

[B65] Palmenberg A. C. Gern J. E. (2015). Classification and evolution of human rhinoviruses. Methods Mol. Biol. 1221, 1–10. 10.1007/978-1-4939-1571-2_1 25261302 PMC4441521

[B66] Pribble C. G. Black P. G. Bosso J. A. Turner R. B. (1990). Clinical manifestations of exacerbations of cystic fibrosis associated with nonbacterial infections. J. Pediatr. 117, 200–204. 10.1016/S0022-3476(05)80530-X 2380817 PMC7130847

[B67] Proud D. Sanders S. P. Wiehler S. (2004). Human rhinovirus infection induces airway epithelial cell production of human beta-defensin 2 both *in vitro* and *in vivo*. J. Immunol. 172, 4637–4645. 10.4049/jimmunol.172.7.4637 15034083

[B68] Rosen B. H. Chanson M. Gawenis L. R. Liu J. Sofoluwe A. Zoso A.. (2018). Animal and model systems for studying cystic fibrosis. J. Cyst. Fibros. 17, S28–S34. 10.1016/j.jcf.2017.09.001 28939349 PMC5828943

[B69] Ruuskanen O. Waris M. Kainulainen L. (2014). Treatment of persistent rhinovirus infection with pegylated interferon α2a and ribavirin in patients with hypogammaglobulinemia. Clin. Infect. Dis. 58, 1784–1786. 10.1093/cid/ciu169 24633687 PMC7108044

[B70] Safari-Alighiarloo N. Taghizadeh M. Tabatabaei S. M. Shahsavari S. Namaki S. Khodakarim S.. (2016). Identification of new key genes for type 1 diabetes through construction and analysis of the protein-protein interaction networks based on blood and pancreatic islet transcriptomes. J. Diabetes 9, 764–777. 10.1111/1753-0407.12483 27625010

[B71] Sanders S. P. Siekierski E. S. Porter J. D. Richards S. M. Proud D. (1998). Nitric oxide inhibits rhinovirus-induced cytokine production and viral replication in a human respiratory epithelial cell line. J. Virol. 72, 934–942. 9444985 10.1128/jvi.72.2.934-942.1998 PMC124563

[B72] Schögler A. Kopf B. S. Edwards M. R. Johnston S. L. Casaulta C. Kieninger E.. (2014). Novel antiviral properties of azithromycin in cystic fibrosis airway epithelial cells. Eur. Respir. J. 45, 428–439. 10.1183/09031936.00102014 25359346

[B73] Sessions O. M. Tan Y. Goh K. C. Liu Y. Tan P. Rozen S.. (2013). Host cell transcriptome profile during wild-type and attenuated dengue virus infection. PLoS Negl. Trop. Dis. 7:e2107. 10.1371/journal.pntd.0002107 23516652 PMC3597485

[B74] Shah A. Connelly M. Whitaker P. McIntyre C. Etherington C. Denton M.. (2015). Pathogenicity of individual rhinovirus species during exacerbations of cystic fibrosis. Eur. Respir. J. 45, 1748–1751. 10.1183/09031936.00229114 25976682

[B75] Sirota M. Dudley J. T. Kim J. Chiang A. P. Morgan A. A. Sweet-Cordero A.. (2011). Discovery and preclinical validation of drug indications using compendia of public gene expression data. Sci. Transl. Med. 3:96ra77. 10.1126/scitranslmed.3003215 21849665 PMC3502016

[B76] Sly P. D. Gangell C. L. Chen L. Ware R. S. Ranganathan S. Mott L. S.. (2013). Risk factors for bronchiectasis in children with cystic fibrosis. N. Engl. J. Med. 368, 1963–1970. 10.1056/NEJMoa1301725 23692169

[B77] Smyth A. R. Smyth R. L. Tong C. Y. Hart C. A. Heaf D. P. (1995). Effect of respiratory virus infections including rhinovirus on clinical status in cystic fibrosis. Arch. Dis. Child. 73, 117–120. 7574853 10.1136/adc.73.2.117 PMC1511210

[B78] Sperber S. J. Levine P. A. Innes D. J. Mills S. E. Hayden F. G. (1988). Tolerance and efficacy of intranasal administration of recombinant β(serine) interferon in healthy adults. J. Infect. Dis. 158, 166–175. 10.1093/infdis/158.1.166 2839579

[B79] Stein R. A. (2017). Hopes and challenges for the common cold. Int. J. Clin. Pract. 71:e12921. 10.1111/ijcp.12921 28238227

[B80] Stelzer-Braid S. Johal H. Skilbeck K. Steller A. Alsubie H. Tovey E.. (2012). Detection of viral and bacterial respiratory pathogens in patients with cystic fibrosis. J. Virol. Methods 186, 109–112. 10.1016/j.jviromet.2012.08.008 22940004

[B81] Stelzer-Braid S. Liu N. Doumit M. D'Cunha R. Belessis Y. Jaffe A.. (2017). Association of rhinovirus with exacerbations in young children affected by cystic fibrosis: preliminary data. J. Med. Virol. 89, 1494–1497. 10.1002/jmv.24794 28213960

[B82] Sutanto E. N. Kicic A. Foo C. J. Stevens P. T. Mullane D. Knight D. A.. (2011). Innate inflammatory responses of pediatric cystic fibrosis airway epithelial cells: effects of nonviral and viral stimulation. Am. J. Respir. Cell Mol. Biol. 44, 761–767. 10.1165/rcmb.2010-0368OC 21317379

[B83] Tacon C. E. Wiehler S. Holden N. S. Newton R. Proud D. Leigh R. (2010). Human rhinovirus infection up-regulates MMP-9 production in airway epithelial cells via NF-{kappa}B. Am. J. Respir. Cell Mol. Biol. 43, 201–209. 10.1165/rcmb.2009-0216OC 19783786

[B84] Turner R. B. Wecker M. T. Pohl G. Witek T. J. McNally E. St George R.. (1999). Efficacy of tremacamra, a soluble intercellular adhesion molecule 1, for experimental rhinovirus infection: a randomized clinical trial. JAMA 281, 1797–1804. 10340366 10.1001/jama.281.19.1797

[B85] Unger B. L. Ganesan S. Comstock A. T. Faris A. N. Hershenson M. B. Sajjan U. S. (2014). Nod-Like Receptor X-1 Is Required for Rhinovirus-Induced Barrier Dysfunction in Airway Epithelial Cells. J. Virol. 88, 3705–3718. 10.1128/JVI.03039-13 24429360 PMC3993547

[B86] van Ewijk B. E. van der Zalm M. M. Wolfs T. F. W. van der Ent C. K. (2005). Viral respiratory infections in cystic fibrosis. J. Cyst. Fibros. 4(Suppl. 2), 31–36. 10.1016/j.jcf.2005.05.011 15964785 PMC7105219

[B87] Vareille M. Kieninger E. Edwards M. R. Regamey N. (2011). The airway epithelium: soldier in the fight against respiratory viruses. Clin. Microbiol. Rev. 24, 210–229. 10.1128/CMR.00014-10 21233513 PMC3021210

[B88] Vlaic S. Conrad T. Tokarski-Schnelle C. Gustafsson M. Dahmen U. Guthke R.. (2018). ModuleDiscoverer: identification of regulatory modules in protein-protein interaction networks. Sci. Rep. 8:433. 10.1038/s41598-017-18370-2 29323246 PMC5764996

[B89] Wagener A. H. Zwinderman A. H. Luiten S. Fokkens W. J. Bel E. H. Sterk P. J.. (2014). dsRNA-induced changes in gene expression profiles of primary nasal and bronchial epithelial cells from patients with asthma, rhinitis and controls. 15:9. 10.1186/1465-9921-15-9 24475887 PMC3916078

[B90] Wark P. A. B. Tooze M. Cheese L. Whitehead B. Gibson P. G. Wark K. F.. (2012). Viral infections trigger exacerbations of cystic fibrosis in adults and children. Eur. Respir. J. 40, 510–512. 10.1183/09031936.00202311 22855475

[B91] Wat D. (ed.). (2015). Respiratory virus in cystic fibrosis – a review of the literature, in Cystic Fibrosis in the Light of New Research (InTechOpen), 143–169. 10.5772/60905

[B92] Wat D. Gelder C. Hibbitts S. Cafferty F. Bowler I. Pierrepoint M.. (2008). The role of respiratory viruses in cystic fibrosis. J. Cyst. Fibros. 7, 320–328. 10.1016/j.jcf.2007.12.002 18255355 PMC7105190

[B93] Waters V. Ratjen F. (2015). Pulmonary exacerbations in children with cystic fibrosis. Ann. Am. Thorac Soc. 12 (Suppl 2), S200–S206. 10.1513/AnnalsATS.201502-098AW 26595740

[B94] Whitsett J. A. Alenghat T. (2015). Respiratory epithelial cells orchestrate pulmonary innate immunity. Nat. Immunol. 16, 27–35. 10.1038/ni.3045 25521682 PMC4318521

[B95] Worrall G. (2011). Common cold. Can. Fam. Physician 57, 1289–1290. 22084460 PMC3215607

[B96] Yeo N.-K. Jang Y. J. (2010). Rhinovirus infection-induced alteration of tight junction and adherens junction components in human nasal epithelial cells. Laryngoscope 120, 346–352. 10.1002/lary.20764 20013846

[B97] Zaman N. Li L. Jaramillo M. L. Sun Z. Tibiche C. Banville M.. (2013). Signaling network assessment of mutations and copy number variations predict breast cancer subtype-specific drug targets. Cell Rep. 5, 216–223. 10.1016/j.celrep.2013.08.028 24075989

